# NR1D1‐transactivated lncRNA NUTM2A‐AS1 promotes chemoresistance and immune evasion in neuroblastoma via inhibiting B7‐H3 degradation

**DOI:** 10.1111/jcmm.18360

**Published:** 2024-05-24

**Authors:** Tian Xiang, Yejing Li, Gao Liu, Xianyun Li

**Affiliations:** ^1^ Department of Clinical Laboratory Center Central Hospital of Enshi Tujia and Miao Autonomous Prefecture Enshi China; ^2^ Department of Gastrointestinal Surgery Central Hospital of Enshi Tujia and Miao Autonomous Prefecture Enshi China

**Keywords:** B7‐H3, cisplatin, neuroblastoma, NR1D1, NUTM2A‐AS1

## Abstract

Neuroblastoma (NB), a common solid tumour in young children originating from the sympathetic nervous system during embryonic development, poses challenges despite therapeutic advances like high‐dose chemotherapy and immunotherapy. Some survivors still grapple with severe side effects and drug resistance. The role of lncRNA NUTM2A‐AS1 has been explored in various cancers, but its function in drug‐resistant NB progression is unclear. Our study found that NUTM2A‐AS1 expression in cisplatin‐resistant NB cells increased in a time‐ and dose‐dependent manner. Knockdown of NUTM2A‐AS1 significantly improved NB cell sensitivity to cisplatin and inhibited metastatic abilities. Additionally, we identified B7‐H3, an immune checkpoint‐related protein, as a NUTM2A‐AS1‐associated protein in NB cells. NUTM2A‐AS1 was shown to inhibit the protein degradation of B7‐H3. Moreover, NUTM2A‐AS1 modulated immune evasion in cisplatin‐resistant NB cells through B7‐H3. Furthermore, NUTM2A‐AS1 expression in cisplatin‐resistant NB cells was transactivated by NR1D1. In summary, our results unveil the molecular or biological relationship within the NR1D1/NUTM2A‐AS1/B7‐H3 axis in NB cells under cisplatin treatment, providing an intriguing avenue for fundamental research into cisplatin‐resistant NB.

## 1 INTRODUCTION

Neuroblastoma (NB) is a solid tumour originating from neural crest tissue and constitutes approximately 15% of tumour‐related deaths in children.^1^ This malignancy displays significant heterogeneity, with high‐risk patients having a survival rate of less than 50%, even with multimodal therapy. Remarkably, certain patients undergo spontaneous tumour regression with minimal or no therapeutic intervention.^2^ NB poses therapeutic challenges with patients categorized into low‐risk, intermediate‐risk and high‐risk subgroups based on factors such as disease stage, age at diagnosis and non‐random chromosomal aberrations.^3^ Notably, a significant proportion of low‐risk patients undergo spontaneous regression, whereas those in the high‐risk subgroups face a survival rate of only 50%, even with aggressive therapeutic interventions.^4^ Cisplatin, also known as cis‐diamminedichloroplatinum (CDDP), represents a platinum coordination compound widely recognized for its effectiveness in chemotherapy. It is commonly used alone or in combination with other agents, such as cyclophosphamide, doxorubicin or etoposide, for the treatment of NB.^5^ Functioning as a cytotoxic drug, CDDP exerts its anti‐cancer effects by causing DNA damage and inhibiting DNA synthesis.^6^, ^7^ Despite its high efficacy, cancer cells frequently develop resistance to CDDP, posing a challenge in its clinical application.

In recent decades, a growing body of evidence has illuminated the crucial roles played by the non‐coding portion of the genome in various cancers. Long non‐coding RNA (lncRNA), a subclass of RNA transcripts characterized by lengths exceeding 200 nucleotides and lacking protein‐coding potential, has emerged as a significant player in the intricate regulatory networks of cellular processes.^8^ Notably, by engaging in interactions with proteins, lncRNAs orchestrate the intricate regulation of gene expression. This regulation occurs at diverse levels, including transcriptional, post‐translational and translational processes, as well as influencing protein activation or degradation.^9^ In the realm of NB, studies have revealed that the dysregulated expression of specific lncRNAs can disrupt processes such as cell proliferation, apoptosis, migration, invasion and even the initiation and progression of tumours.^10^, ^11^, ^12^ Moreover, emerging evidence suggests that lncRNAs might exert significant influences on determining the sensitivity of neuroblastoma to chemotherapy.^13^, ^14^ However, the precise mechanisms through which long non‐coding RNAs (lncRNAs) influence chemotherapy in NB and their potential role in conferring resistance to the anticancer effects of chemotherapy drugs remain unexplored and warrant further investigation.

NUTM2A‐AS1, belonging to the lncRNA family, has been identified as an oncogene in non‐small cell lung cancer (NSCLC) and gastric cancer (GC).^15^, ^16^ Moreover, its significant upregulation and association with poor prognosis in hepatocellular carcinoma (HCC).^17^ Despite these findings, the precise mechanism of action of lncRNA NUTM2A‐AS1 in NB remains unclear.

Therefore, we conducted a comprehensive investigation in this study. Our investigation revealed that the expression of NUTM2A‐AS1 increased in cisplatin‐resistant NB cells in a time‐ and dose‐dependent manner. Knockdown of NUTM2A‐AS1 significantly enhanced the sensitivity of NB cells to cisplatin and attenuated metastatic abilities. Furthermore, we identified B7‐H3, an immune checkpoint‐related protein, as a NUTM2A‐AS1‐associated protein in NB cells. NUTM2A‐AS1 was found to inhibit the protein degradation of B7‐H3. Additionally, NUTM2A‐AS1 played a role in modulating immune evasion in cisplatin‐resistant NB cells through B7‐H3. Notably, NUTM2A‐AS1 expression in cisplatin‐resistant NB cells was revealed to be transactivated by nuclear receptor subfamily 1 group d member 1 (NR1D1). In summary, our findings elucidate the molecular and biological interplay within the NR1D1/NUTM2A‐AS1/B7‐H3 axis in NB cells under cisplatin treatment, offering a promising avenue for fundamental research on cisplatin‐resistant NB.

## 2 MATERIALS AND METHODS

### 2.1 Cell Culture

The neuroblastoma cell lines (SK‐N‐SH and SHSY‐5Y) were obtained from the American Type Culture Collection (ATCC). All cells were cultured in Dulbecco's modified Eagle's medium (DMEM; Gibco) supplemented with 10% fetal bovine serum (FBS; Gibco) and maintained at 37°C in a 5% CO_2_ humidified environment.

To establish cisplatin‐resistant cells (SHSY‐5Y‐R and SK‐N‐SH‐R cells), SHSY‐5Y and SK‐N‐SH cells were exposed to gradually increasing concentrations of cisplatin over a 6‐month period.

### 2.2 Reverse transcription‐quantitative polymerase chain reaction (RT‐qPCR)

Total RNA was extracted using TRIzol reagent (Invitrogen), and its concentration and purity were assessed spectrophotometrically. The purified RNA was subjected to reverse transcription using the GoScript Reverse Transcription System (Qiagen GmbH) to synthesize complementary DNA (cDNA). RT‐qPCR was carried out with the One Step SYBR RT‐PCR Kit (TaKaRa) on a Roche LightCycler 480 (Roche), involving an initial denaturation at 95°C for 30 s, followed by 40 cycles of denaturation at 95°C for 5 s, annealing at 55°C for 30 s and extension at 72°C for 30 s. Gene expression levels were normalized to glyceraldehyde‐3‐phosphate dehydrogenase (GAPDH) and U6 small nuclear RNA. The relative gene expression was calculated using the 2^−ΔΔCt^ method. The primer sequences are listed in Table 1.

**TABLE 1:** Primer sequences in qRT‐PCR assay.

Gene	Primer
NUTM2A‐AS1	GCCTCCAGTTCTTAGAAT (forward; F)
	ATTCTAAGAACTGGAGGC (reverse; R)
NR1D1	TGGACTCCAACAACAACACAG (forward; F)
	GATGGTGGGAAGTAGGTGGG (reverse; R)
B7‐H3	ACAGGGCAGCCTATGACATT (forward; F)
	CTGCATTCTCCTCCTCACAG (reverse; R)
GAPDH	TGTGGGCATCAATGGATTTGG (forward; F)
	ACACCATGTATTCCGGGTCAAT (reverse; R)

### 2.3 Western blot

Proteins were extracted with RIPA lysis buffer (Beyotime) and quantified using the bicinchoninic acid method following standard protocols. After blocking with 1% non‐fat milk, immunoblot assays were performed with specific primary antibodies. Subsequently, membranes were incubated with secondary HRP‐conjugated antibodies. Protein signals were visualized using a chemiluminescence chromogenic substrate (Beyotime). Antibodies for western blot analysis are as follows: B7‐H3 (R&D Systems, #AF1027, 1:250) and GAPDH (Abcam, #EPR16891, 1:10000).

### 2.4 Cell proliferation detection

Cell viability and proliferation were assessed using the Cell Counting Kit‐8 (CCK‐8) assay kit (Dojondo). Transfected cells (3000 cells per well) were seeded into 96‐well plates and incubated for 48 h. Subsequently, 10 μL of CCK‐8 solution was added to each well, and the absorbance at 450 nm was measured using a microplate reader (Bio‐Rad) after further incubation at 37°C with 5% CO_2_ for 2 h. The half maximal inhibitory concentration (IC50) value of cisplatin was determined based on the relative survival curve.

For the examination of cell growth, a colony formation assay was performed. Cells were plated in 6‐well plates at a density of 600 cells/well and cultured for 14 days. The cell colonies were fixed with 4% paraformaldehyde, stained with 0.1% crystal violet and counted under a microscope.

### 2.5 Cell migration and invasion detection

The migration and invasion abilities of cells were assessed using 24‐well Transwell plates (Corning). In brief, the upper chamber of the Transwell chamber, pre‐coated with Matrigel for invasion assays or left uncoated for migration assays, received 200 μL of serum‐free medium containing 2 × 104 cells. Simultaneously, the lower chamber was filled with 600 μL of complete medium containing 10% FBS as a chemoattractant. After 48 h of incubation, the migrated or invaded cells were fixed with 4% paraformaldehyde, stained with 0.5% crystal violet and counted using a microscope (Zeiss).

### 2.6 RNA pull‐down assays and mass spectrometry analysis

Cell lysates were resuspended in pull‐down lysis buffer (20 mM Tris, pH 7.5, 150 mM NaCl, 1.5 mM MgCl2, 10% glycerol, 0.5% NP‐40, 0.5% TritonX‐100, phosphatase inhibitors, protease inhibitors cocktail and RNase inhibitor). The homogenized suspension was then centrifuged at 12,000 rpm for 15 min, and the resulting supernatants were incubated with 400 pmol of biotin‐labelled DNA probes, 2 μg of in vitro circularized circRNAs or 200 pmol of biotin‐labelled RNA probes at 4°C for 4 h.

Following probe incubation, the lysates were further incubated with 20 μL of prepared streptavidin magnetic beads (Thermo, #65601) for an additional 1 h at 4°C, followed by five washes with wash buffer (20 mM Tris–HCl, pH 7.5, 1 mM EDTA and 300 mM NaCl). The pulled‐down proteins were then subjected to either mass spectrometry or western blot analysis.

For mass spectrometry, immunoprecipitated proteins underwent short‐gel sodium dodecyl sulfate–polyacrylamide gel electrophoresis for 20 min. The protein bands of interest were excised, washed and dehydrated with acetonitrile. Subsequently, 10 mmol/L dithiothreitol (Sigma‐Aldrich, #D0632) was applied for reduction, and 55 mmol/L iodoacetamide (Sigma‐Aldrich, #V900335) for alkylation. Proteins were then digested with trypsin (Promega, #V5071) for 12 h. After desalting using C18 ZipTip (Millipore) following the manufacturer's instructions, peptide samples were analysed by LC–MS/MS.

### 2.7 RNA immunoprecipitation (RIP) assay

The RIP assay was conducted using the Magna RNA immunoprecipitation kit (Millipore). Neuroblastoma cells were lysed, and 100 μL of lysate was incubated with magnetic beads coated with IgG antibody. Subsequently, the immunoprecipitated RNA was extracted and purified, and the RNA was analysed by qRT‐PCR using the 7500 Real‐Time PCR system (Thermo Fisher Scientific).

### 2.8 Chromatin immunoprecipitation (ChIP) assay

ChIP assay was conducted using a ChIP kit from Merck Millipore following the manufacturer's instructions. Subsequently, qPCR analysis was performed to detect the DNA fragments that co‐immunoprecipitated with NR1D1.

### 2.9 Immunoprecipitation (IP) and ubiquitination assays

IP and ubiquitination assays were performed using the Thermo Scientific Pierce Classic IP kit (Thermo Fisher Scientific) following the manufacturer's instructions. NB cells with indicated transfections were treated with or without MG‐132 (the inhibitor of proteasome) for 24 h. Subsequently, cells were lysed in the IP lysis/wash buffer with protease inhibitors and RNase inhibitor (Promega, Madison, WI., USA) followed by centrifugation. The protein A/G beads (Life Technologies) conjugated with antibodies against B7‐H3 were mixed with cell lysates for 12 h at 4°C with rotation and boiled in the SDS buffer. Thereafter, the eluted proteins were analysed by western blot.

### 2.10 Cytotoxicity assay

NK cell cytotoxicity was assessed by measuring the level of lactate dehydrogenase (LDH) using the CytoTox96 Non‐Radioactive Cytotoxicity Assay Kit (Promega). Briefly, cells were plated at a density of 2 × 10^5 cells/mL into a 96‐well plate. Subsequently, 50 μL of CytoTox96 Reagent was added to each well, and the plate was incubated for 30 min at room temperature. Following this, stop solution (50 μL) was added, and A490 was determined using a microplate reader (Bio‐Rad). Percent cytotoxicity was calculated according to the kit instructions.

### 2.11 ELISA assay

The levels of IFN‐γ and TNF‐α in the cell culture medium were quantified using ELISA kits (BMS228 and BMS223‐4, Invitrogen). Briefly, the culture medium was collected and centrifuged at 1400 rpm for 1 min. The ELISA assay was performed according to the kit instructions, and A450 was determined using a microplate reader (Bio‐Rad).

### 2.12 Luciferase reporter gene assay

Upon achieving an approximate 80% confluence, cells underwent co‐transfection with either wild‐type or mutant versions of the NUTM2A‐AS1 promoter dual‐luciferase reporter vectors and the pcDNA3.1‐NR1D1 plasmids, incorporating the pRL‐TK plasmid as a normalization control. Following a 36 h incubation period, these cells were subsequently harvested for analysis. The subsequent quantification of firefly and Renilla luciferase enzymatic activities was conducted employing the Dual‐Luciferase Reporter Assay System (Promega).

### 2.13 Statistical analysis

Data analysis was conducted using SPSS version 13.0 (SPSS) software. Results are presented as mean ± standard deviation (SD), unless stated otherwise. Statistical methods included one‐way analysis of variance (ANOVA) or Student's *t*‐tests, as appropriate. All experiments were performed in triplicate, and statistical significance was defined as a *p*‐value <0.05.

## 3 RESULTS

### 3.1 NUTM2A‐AS1 is upregulated in cisplatin resistant neuroblastoma cells and regulates NB cell sensitivity to cisplatin

**FIGURE 1:**
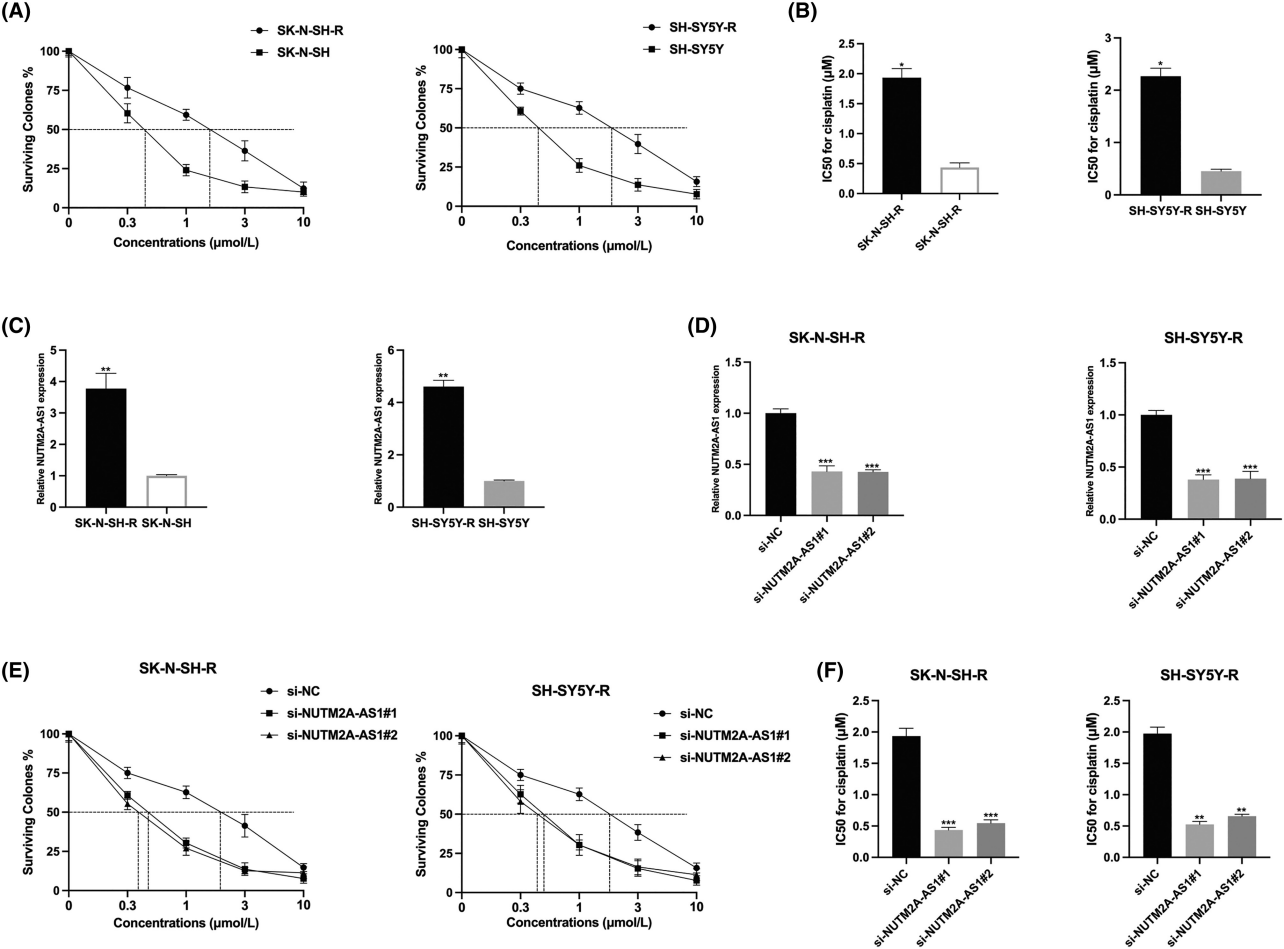
NUTM2A‐AS1 is upregulated in cisplatin resistant neuroblastoma cells and regulates neuroblastoma (NB) cell sensitivity to cisplatin. (A,B) The Cell Counting Kit‐8 (CCK‐8) assay was employed to determine the IC50 value of SH‐SY5Y and SH‐SY5Y‐R, as well as SK‐N‐SH and SK‐N‐SH‐R NB cells, respectively. (C) A quantitative real‐time polymerase chain reaction (qRT‐PCR) assay was conducted to assess the expression levels of NUTM2A‐AS1 in both SH‐SY5Y and SH‐SY5Y‐R, as well as SK‐N‐SH and SK‐N‐SH‐R NB cells. (D) NUTM2A‐AS1 knockdown models were established by transfecting SH‐SY5Y‐R and SK‐N‐SH‐R NB cells with si‐NC, si‐NUTM2A‐AS1#1 and si‐NUTM2A‐AS1#2, and the transfection efficiencies were validated using qRT‐PCR. (E,F) The Cell Counting Kit‐8 (CCK‐8) assay was subsequently employed to assess the IC50 value of NUTM2A‐AS1 knockdown in SH‐SY5Y‐R and SK‐N‐SH‐R NB cells. *p < 0.05, **p < 0.01, ***p < 0.001.

In an endeavour to discern the involvement of NUTM2A‐AS1 in cisplatin‐resistant NB progression, we initially cultivated cisplatin‐resistant NB cell lines. The CCK‐8 assay demonstrated that the IC50 values for cisplatin in SH‐SY5Y‐R and SK‐N‐SH‐R cells were significantly higher than those in their parental counterparts, SH‐SY5Y and SK‐N‐SH (Figure 1A,B), signifying the successful creation of cisplatin‐resistant cells. Moreover, an upsurge in NUTM2A‐AS1 expression was observed in the SH‐SY5Y‐R and SK‐N‐SH‐R cells in comparison to the SH‐SY5Y and SK‐N‐SH cells (Figure 1C), implying a possible role of NUTM2A‐AS1 in the resilience of NB to cisplatin. Subsequent knockdown of NUTM2A‐AS1 in the resistant cells led to a notable decrease in NUTM2A‐AS1 levels (Figure 1D). Post‐knockdown, the CCK‐8 assay indicated a significant reduction in the IC50 values of cisplatin in NUTM2A‐AS1 silenced SH‐SY5Y‐R and SK‐N‐SH‐R cells compared to the original resistant models (Figure 1E,F), reinforcing the hypothesis that NUTM2A‐AS1 functions pivotally in cisplatin‐resistant NB.

### 3.2 NUTM2A‐AS1 silence inhibits NB cell viability and metastasis

**FIGURE 2:**
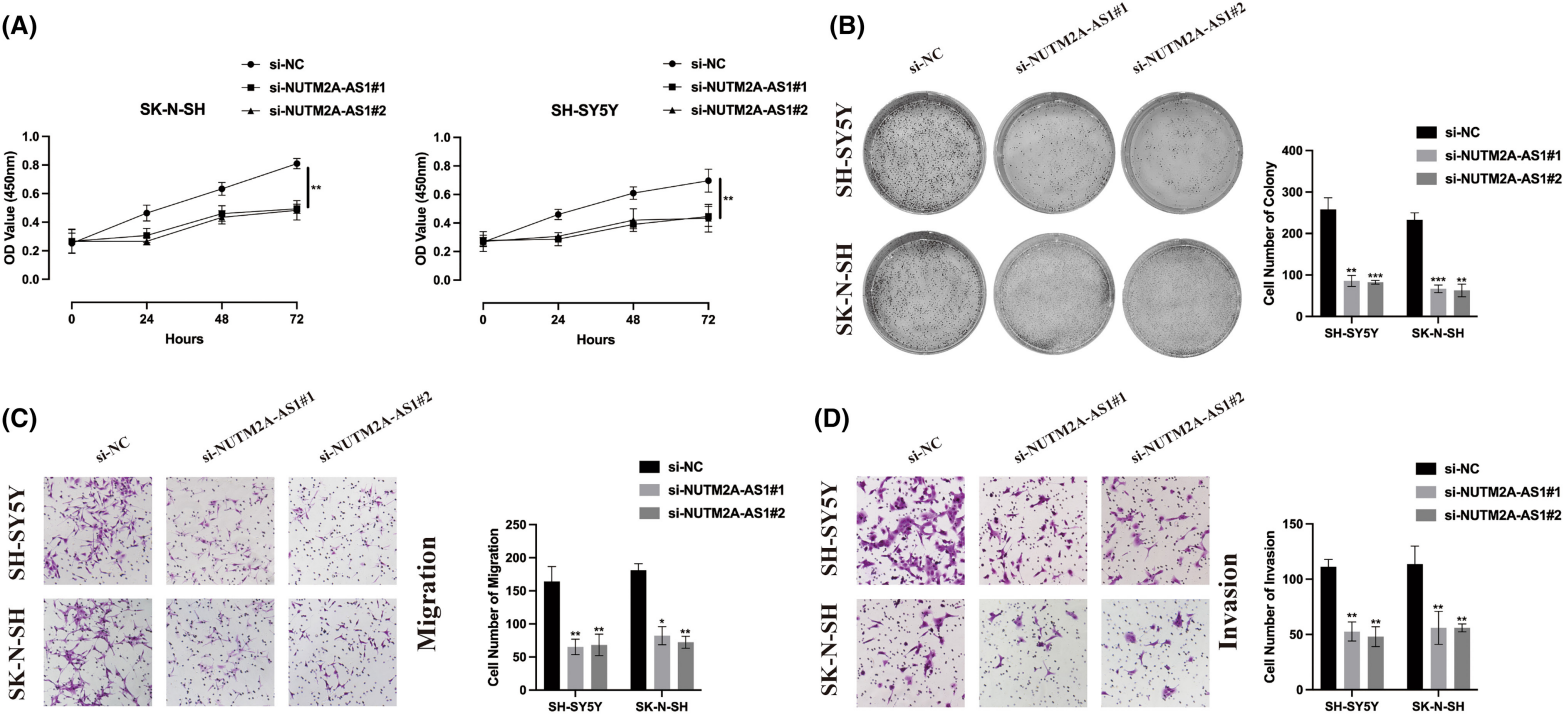
NUTM2A‐AS1 silence inhibits neuroblastoma (NB) cell viability and metastasis. (A) Cell Counting Kit‐8 (CCK‐8) assay was employed to assess the proliferation level of NB cells following NUTM2A‐AS1 knockdown. (B) The cell colony formation assay was utilized to evaluate the proliferation ability of NB cells with depleted NUTM2A‐AS1. (C) The Transwell migration assay was conducted to investigate the migration ability of NB cells upon NUTM2A‐AS1 knockdown. (D) The Transwell Invasion assay was performed to measure the invasion ability of NB cells following NUTM2A‐AS1 depletion. *p < 0.05, **p < 0.01, ***p < 0.001.

The function of NUTM2A‐AS1 was further examined in standard NB cell lines. Utilizing CCK‐8 and cell colony formation assays, a notable suppression in NB cell proliferation was observed following the knockdown of NUTM2A‐AS1, as depicted in Figure 2A,B. Additionally, the outcomes of Transwell migration (Figure 2C) and invasion (Figure 2D) assays indicated a significant reduction in the metastatic potential of NB cells upon NUTM2A‐AS1 silencing. These results collectively suggest that NUTM2A‐AS1 operates as an oncogene in the progression of NB, highlighting its influence on both cell proliferation and metastasis.

### 3.3 NUTM2A‐AS1 is associated with B7‐H3 and inhibits B7‐H3 ubiquitination level

**FIGURE 3:**
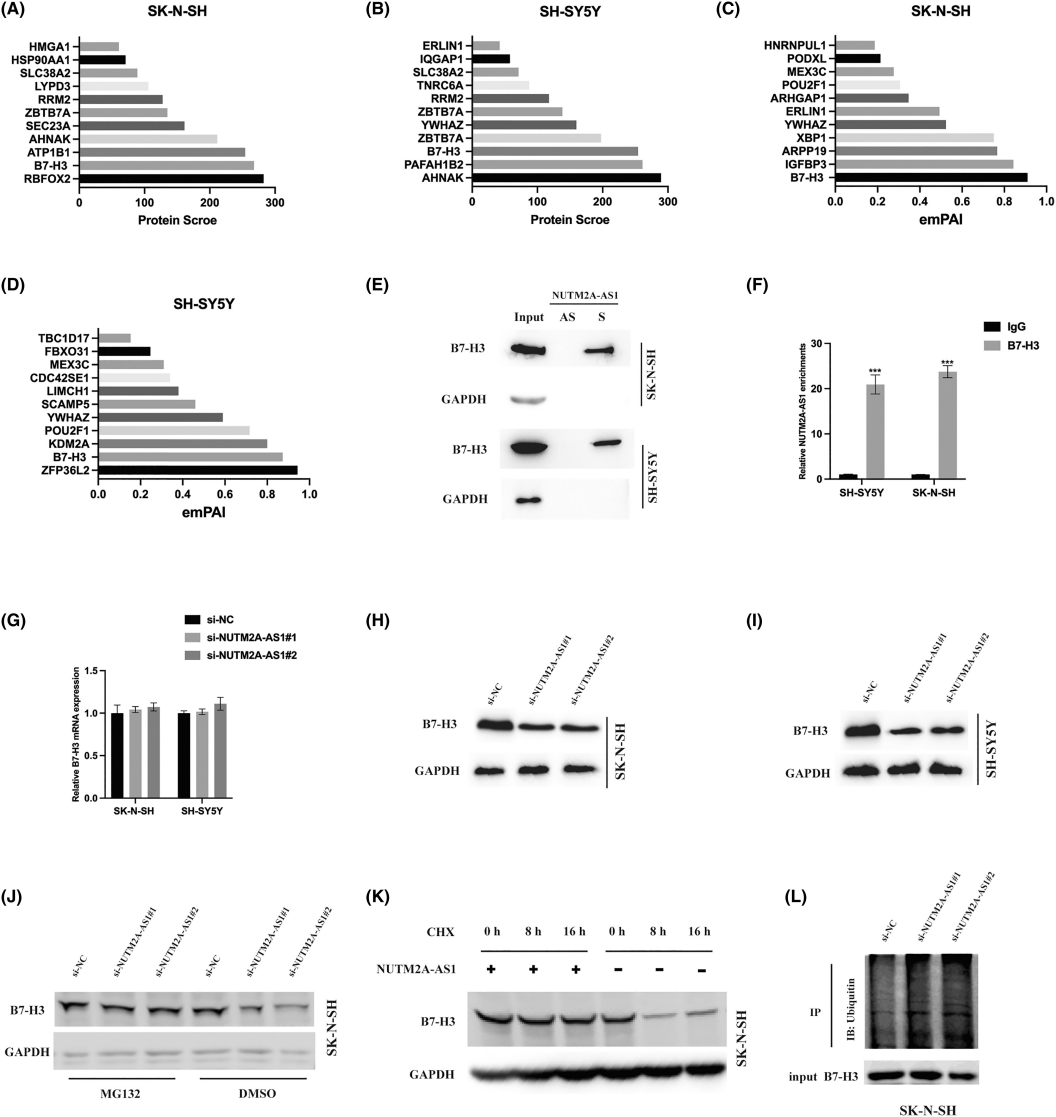
NUTM2A‐AS1 is associated with B7‐H3 and inhibits B7‐H3 ubiquitination level. (A,B) The graphs depict the protein scores of the 11 proteins identified through RNA pull‐down and LC–MS analysis. (C,D) The graphs illustrate the exponentially modified protein abundance index (emPAI) of the 11 proteins identified by RNA pull‐down assays and LC–MS. (E) B7‐H3 was specifically pulled down by biotin‐labelled sense NUTM2A‐AS1 (S) but not NUTM2A‐AS1 anti‐sense (AS) RNA in the indicated cells. (F) RNA immunoprecipitation (RIP) assays were conducted using anti‐B7‐H3 antibodies with extracts from neuroblastoma (NB) cells. Relative enrichment represents the RNA levels associated with B7‐H3 relative to an input control, comparing anti‐B7‐H3 antibody immunoprecipitation with IgG antibody. GAPDH mRNA served as the negative control. (G) B7‐H3 mRNA expression in NUTM2A‐AS1 knockdown cells was assessed by qRT‐PCR assay. (H,I) B7‐H3 protein expression in NUTM2A‐AS1 knockdown cells was examined by western blot assay. (J) NB cells with NUTM2A‐AS1 knockdown and control cells were treated with or without MG132 (5 μM) for 12 h, and cell lysates were analysed by western blotting. (K) NB cells with or without siRNAs specific for NUTM2A‐AS1 were treated with 20 μg/mL cycloheximide (CHX) or a vehicle for the indicated periods, and B7‐H3 levels were analysed by western blotting. (L) Western blot analysis of the ubiquitination of B7‐H3 in NUTM2A‐AS1 knockdown SK‐N‐SH cells. ***p < 0.001.

To elucidate the molecular underpinnings of NUTM2A‐AS1's role in NB, we undertook RNA pull‐down assays coupled with MS analysis to identify proteins interacting with NUTM2A‐AS1. Analysis of SH‐SY5Y‐R/SK‐N‐SH‐R cells relative to controls yielded 11 candidate proteins based on their scores and the exponentially modified protein abundance index (emPAI) derived from MS data. B7‐H3 was selected for further investigation (Figure 3A–D). Subsequent RNA pull‐down and RIP assays verified the direct interaction between NUTM2A‐AS1 and B7‐H3 in NB cells (Figure 3E,F). Interestingly, while NUTM2A‐AS1 knockdown diminished B7‐H3 protein levels, mRNA levels remained unaffected (Figure 3G–I). Investigating B7‐H3's stability under NUTM2A‐AS1 modulation, cells with reduced NUTM2A‐AS1 were treated with proteasome inhibitor MG132, which did not lead to a decrease in endogenous B7‐H3 levels (Figure 3J). Conversely, elevating NUTM2A‐AS1 expression and applying the protein synthesis inhibitor cycloheximide (CHX) extended the half‐life of B7‐H3 compared to controls (Figure 3K). Furthermore, immunoprecipitation assays showed increased B7‐H3 ubiquitination in NUTM2A‐AS1 knockdown cells (Figure 3L). Collectively, these findings indicate that NUTM2A‐AS1 acts to safeguard B7‐H3 from ubiquitin–proteasome‐mediated degradation, highlighting a novel protective mechanism in NB.

### 3.4 B7‐H3 is upregulated in cisplatin‐resistant neuroblastoma cells and regulates NB cell sensitivity to cisplatin

**FIGURE 4:**
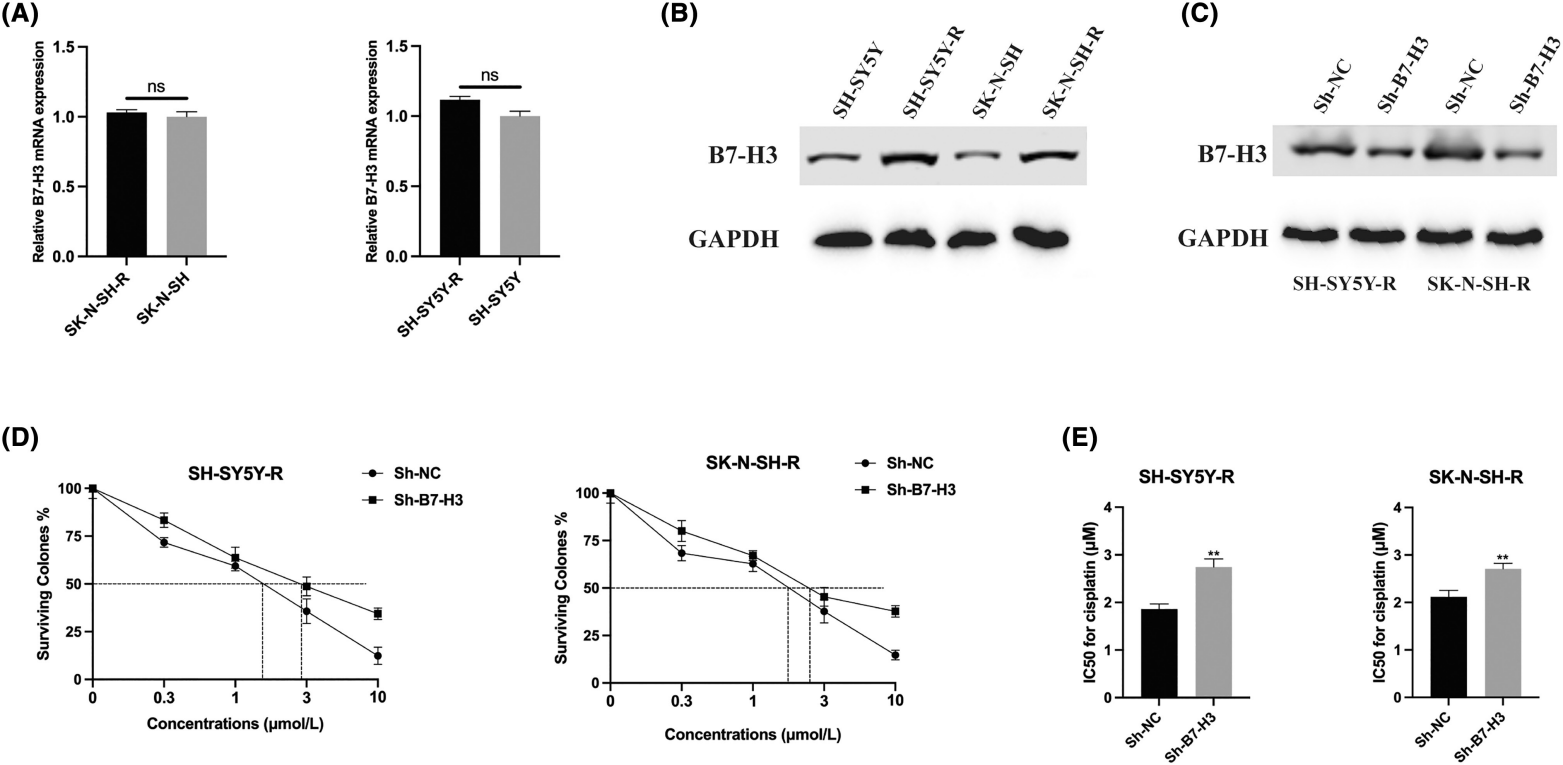
B7‐H3 is upregulated in cisplatin resistant neuroblastoma cells and regulates neuroblastoma (NB) cell sensitivity to cisplatin. (A) B7‐H3 mRNA expression in SH‐SY5Y/SH‐SY5Y‐R and SK‐N‐SH/SK‐N‐SH‐R NB cells was quantified using qRT‐PCR. (B) B7‐H3 protein expression in SH‐SY5Y/SH‐SY5Y‐R and SK‐N‐SH/SK‐N‐SH‐R NB cells was assessed by western blot. (C) B7‐H3 protein expression in B7‐H3 knockdown SH‐SY5Y‐R and SK‐N‐SH‐R NB cells was determined by western blot. (D,E) Cell Counting Kit‐8 (CCK‐8) assay was conducted to determine the IC50 value of B7‐H3 knockdown SH‐SY5Y‐R and SK‐N‐SH‐R NB cells. **p < 0.01.

While numerous investigations have delved into B7‐H3's involvement in neuroblastoma (NB) progression,^18^, ^19^, ^20^ its exact biological function in the context of cisplatin‐resistant NB remains unclear. Our research has uncovered that cisplatin exposure leads to an increase in B7‐H3 protein levels in NB cells without affecting mRNA expression (Figure 4A,B). In cisplatin‐resistant SH‐SY5Y‐R and SK‐N‐SH‐R cells, B7‐H3 expression was effectively reduced (Figure 4C). Subsequent CCK‐8 assays demonstrated a notable decrease in IC50 of cisplatin for B7‐H3 knockdown cells compared to their parental SH‐SY5Y and SK‐N‐SH counterparts (Figure 4D,E), suggesting a pivotal role of B7‐H3 in mediating cisplatin resistance in NB.

### 3.5 B7‐H3 promotes NB cell viability, metastasis and immune escape

**FIGURE 5:**
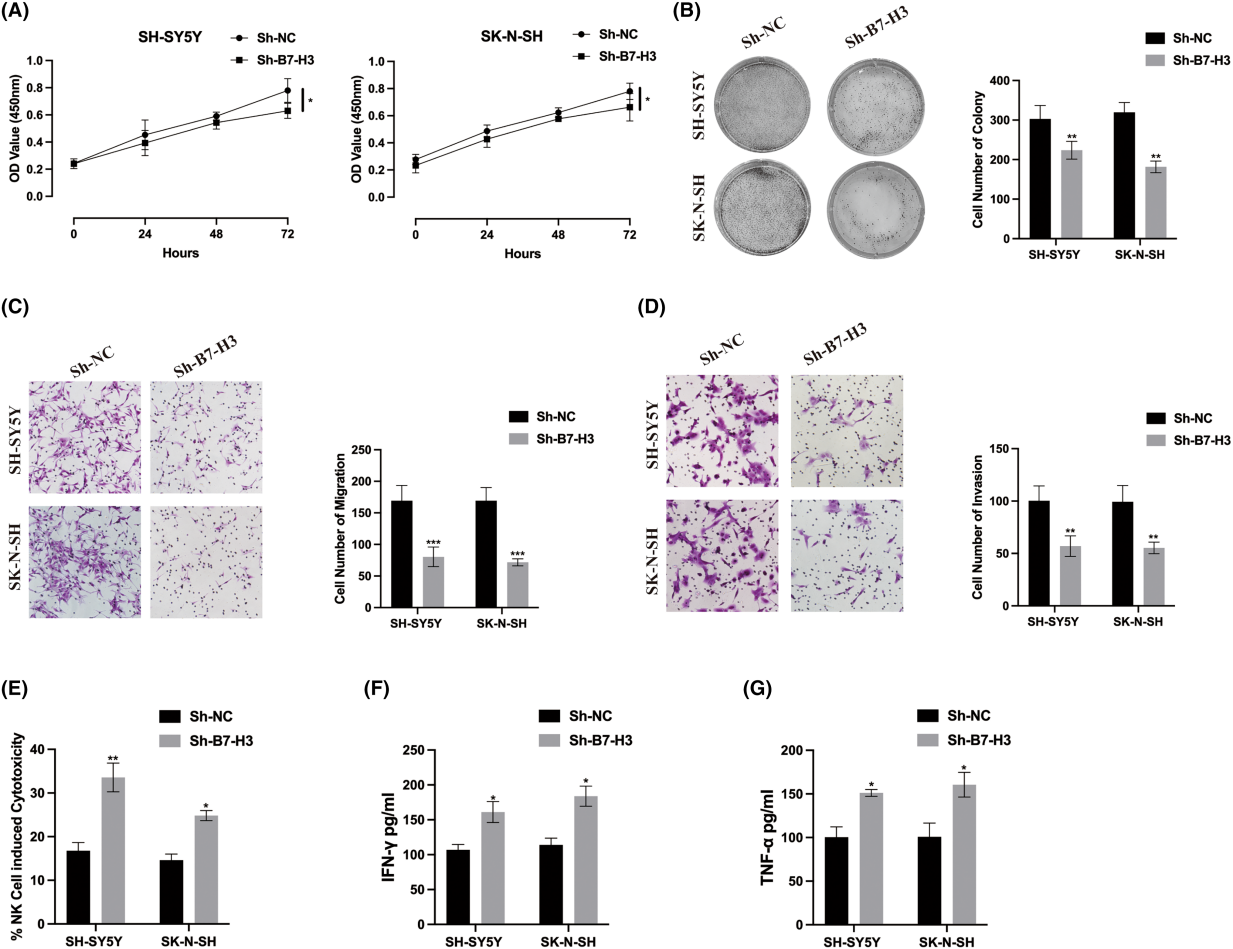
B7‐H3 promotes neuroblastoma (NB) cell viability, metastasis and immune escape. (A) Cell Counting Kit‐8 (CCK‐8) assay was employed to assess the proliferation level of B7‐H3 knockdown NB cells. (B) Cell colony formation assay was conducted to evaluate the proliferation ability of B7‐H3 knockdown NB cells. (C) Transwell migration assay was performed to detect the migration ability of B7‐H3 knockdown NB cells. (D) Transwell invasion assay was employed to measure the invasion ability of B7‐H3 knockdown NB cells. (E) Cytotoxicity of NK92 cells against NB cells was determined by CytoTox96 cytotoxicity assay. (F,G) The levels of secreted IFN‐γ and TNF‐α in the cell culture supernatant of the co‐culture system were measured by ELISA assay. *p < 0.05, **p < 0.01, ***p < 0.001.

The functional impact of B7‐H3 was further evaluated in normal NB cells through CCK‐8 and cell colony assays. As illustrated in Figure 5A,B, the depletion of B7‐H3 significantly inhibited NB cell proliferation. Additionally, transwell migration (Figure 5C) and invasion (Figure 5D) assays revealed a substantial reduction in NB cell metastasis upon B7‐H3 knockdown. These results underscore the oncogenic role of B7‐H3 in NB progression. Previous research has highlighted the crucial role of B7‐H3 in immune escape mechanisms.^21^, ^22^, ^23^, ^24^ Therefore, we investigated whether B7‐H3 affects NB cell immune function. As shown in Figure 5E–G, B7‐H3 knockdown in NB cells heightened the cytotoxicity of NK92 cells, accompanied by an increased secretion of IFN‐γ and TNF‐α. These findings suggest that B7‐H3 acts as an oncogene, suppressing the cytotoxicity of NK92 cells against NB cells.

### 3.6 NUTM2A‐AS1 modulates NB cell sensitivity to cisplatin via B7‐H3

**FIGURE 6:**
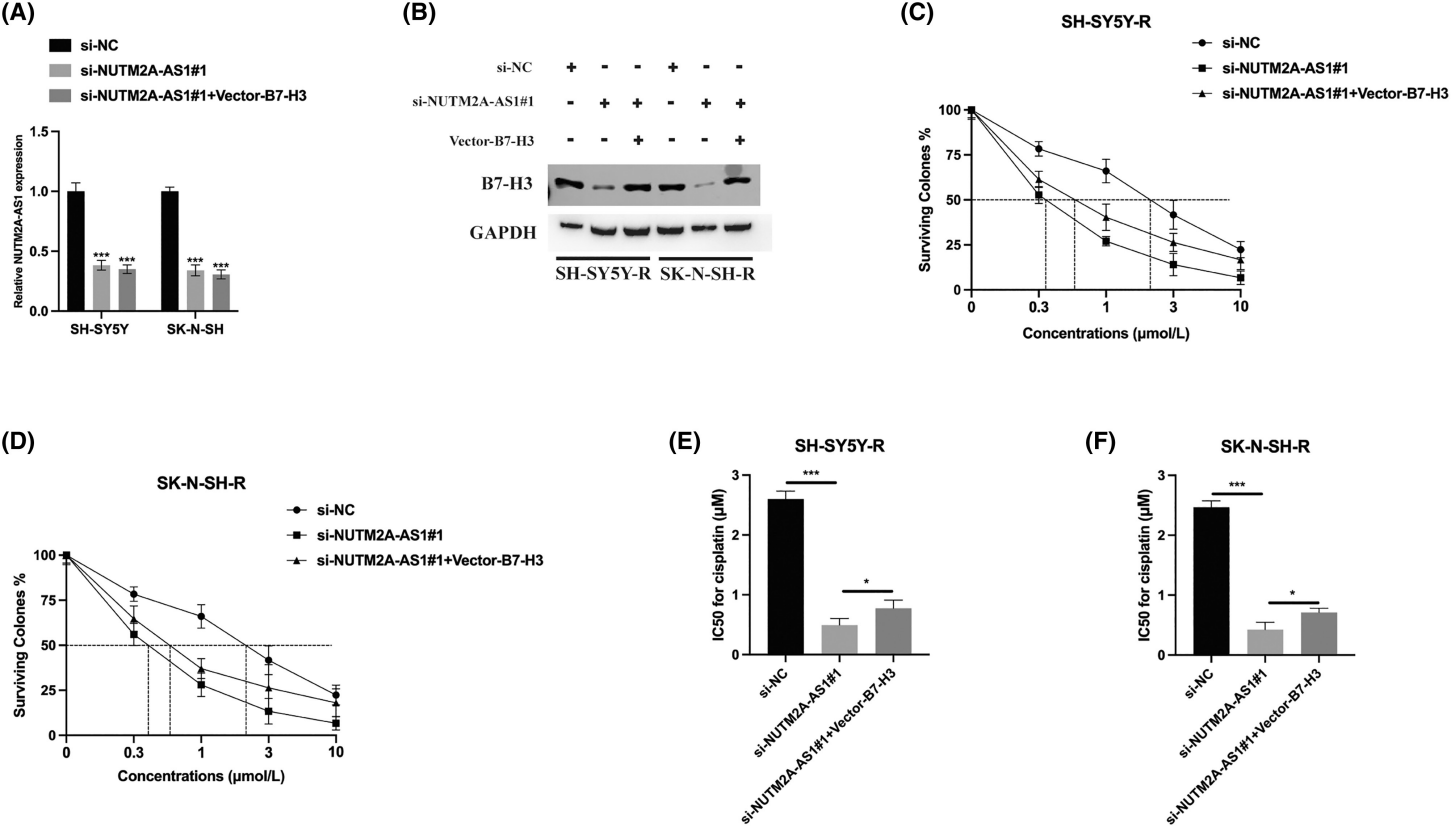
NUTM2A‐AS1 modulates neuroblastoma (NB) cell sensitivity to cisplatin via B7‐H3. (A) NB cells were transfected with si‐NC, si‐NUTM2A‐AS1#1, and Vector‐B7‐H3 and NUTM2A‐AS1 expression was measured by qRT‐PCR assay. (B) Protein expression of B7‐H3 was detected by western blot. (C–F) Cell Counting Kit‐8 (CCK‐8) assay was performed to determine the IC50 value of the indicated SH‐SY5Y‐R and SK‐N‐SH‐R NB cells. *p < 0.05, ***p < 0.001.

Subsequently, we established cell models to confirm the biological link between NUTM2A‐AS1 and B7‐H3. Transfections of both NUTM2A‐AS1 siRNA and B7‐H3 vector were performed in cisplatin‐resistant NB cells, and the results were assessed by qPCR and western blot (Figure 6A,B). CCK‐8 assay results demonstrated that the IC50 value of cisplatin in NUTM2A‐AS1 knockdown SH‐SY5Y‐R and SK‐N‐SH‐R cells was significantly lower than that in SH‐SY5Y and SK‐N‐SH cells. Meanwhile, B7‐H3 overexpression mitigated the impact of NUTM2A‐AS1 knockdown in both SH‐SY5Y‐R and SK‐N‐SH‐R cells. These findings indicate that NUTM2A‐AS1 functions in cisplatin‐resistant NB cells by modulating B7‐H3 expression.

### 3.7 NUTM2A‐AS1 regulates cell viability, metastasis and immune escape through B7‐H3

**FIGURE 7:**
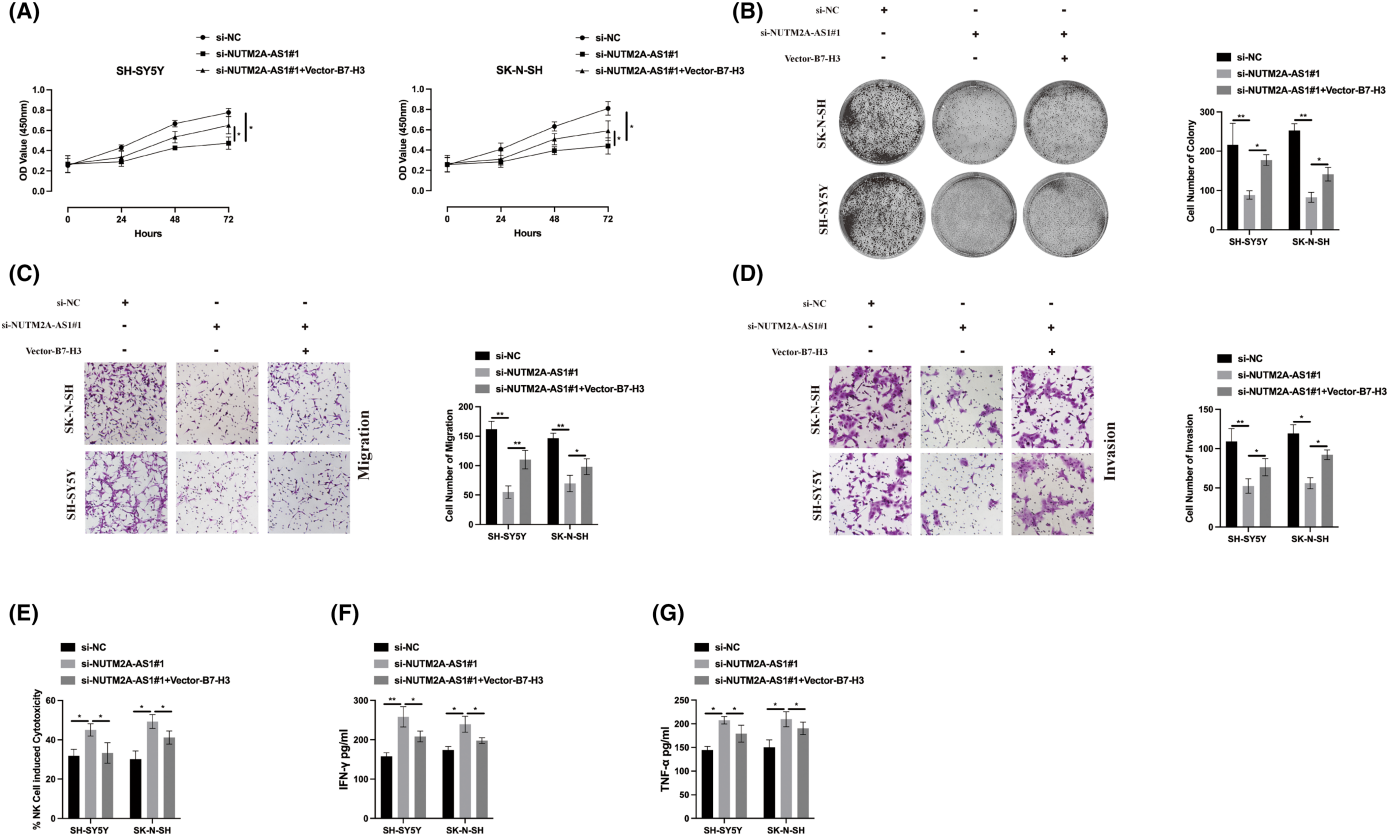
NUTM2A‐AS1 regulates cell viability, metastasis, and immune escape through B7‐H3. (A) The proliferation level of si‐NC, si‐NUTM2A‐AS1#1 and Vector‐B7‐H3 transfected neuroblastoma (NB) cells was assessed using a Cell Counting Kit‐8 (CCK‐8) assay. (B) The proliferation ability of si‐NC, si‐NUTM2A‐AS1#1 and Vector‐B7‐H3 transfected NB cells was evaluated by a cell colony formation assay. (C) The migration ability of si‐NC, si‐NUTM2A‐AS1#1 and Vector‐B7‐H3 transfected NB cells was determined through a Transwell migration assay. (D) The invasion ability of si‐NC, si‐NUTM2A‐AS1#1 and Vector‐B7‐H3 transfected NB cells was measured using a Transwell invasion assay. (E) The cytotoxicity of NK92 cells against transfected NB cells was detected by the CytoTox96 cytotoxicity assay. (F–G) The levels of secreted IFN‐γ and TNF‐α in the cell culture supernatant of the co‐culture system were measured by ELISA assay. *p < 0.05, **p < 0.01, ***p < 0.001.

Our exploration into the NUTM2A‐AS1/B7‐H3 axis's influence on standard NB cells has further delineated its role. The CCK‐8 and cell colony formation assays indicated that overexpressing B7‐H3 could partly mitigate the suppressive impact of NUTM2A‐AS1 knockdown on NB cell viability (Figure 7A,B). This rescue effect was similarly noted in the context of NB cell metastasis; Transwell migration and invasion assays demonstrated that B7‐H3 overexpression reduced the inhibition of cell migration and invasion resulting from NUTM2A‐AS1 knockdown (Figure 7C,D). In terms of immune evasion, the reduction of NUTM2A‐AS1 heightened the cytotoxic response of NK92 cells towards NB cells, evidenced by increased secretion of IFN‐γ and TNF‐α. B7‐H3 overexpression, however, was able to partially counteract the effects of NUTM2A‐AS1 knockdown (Figure 7E–G). These observations highlight the interplay between NUTM2A‐AS1 and B7‐H3 in modulating NB progression, suggesting their potential as targets for therapeutic intervention.

### 3.8 NR1D1‐transactivated NUTM2A‐AS1 expression in NB cells

**FIGURE 8:**
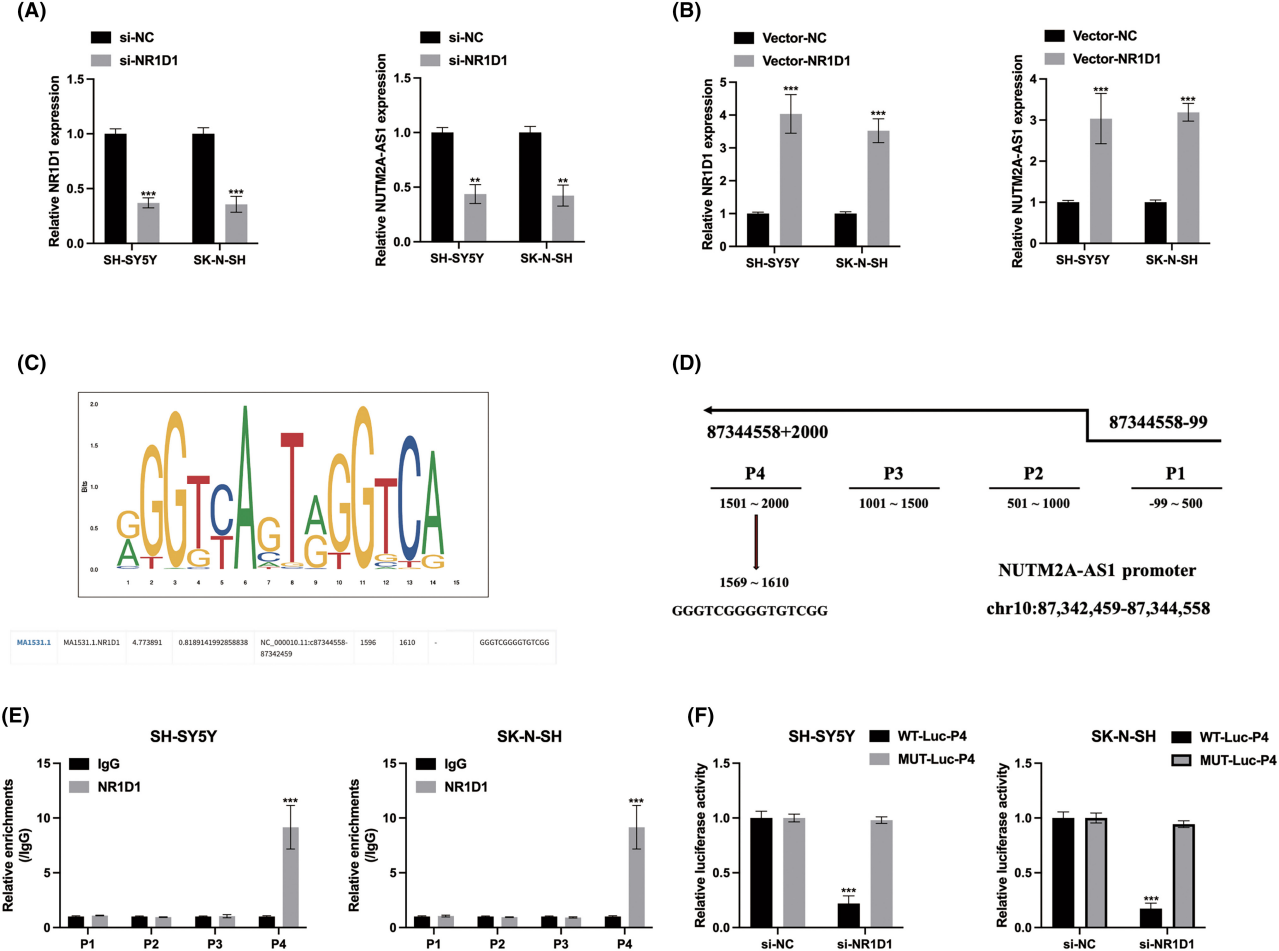
NR1D1‐transactivated NUTM2A‐AS1 expression in neuroblastoma (NB) cells. (A) NB cells were stably transfected with siRNAs targeting NR1D1, and the transfection efficiencies were assessed by qRT‐PCR. (B) NR1D1 vectors were stably introduced into NB cells, and the relative expression of NUTM2A‐AS1 in NR1D1‐overexpressed NB cells was measured by qRT‐PCR. (C) The predicted binding sites of NR1D1 were obtained from the JASPAR dataset. (D) Predicted binding regions on the NUTM2A‐AS1 promoter were acquired from JASPAR. (E) ChIP assay using anti‐IgG and anti‐NR1D1 antibodies was conducted, and the results were analysed using qRT‐PCR. (F) The binding between NR1D1 and the P4 region of NUTM2A‐AS1 was detected via a luciferase reporter gene assay in NB cells. **p < 0.01, ***p < 0.001.

To decipher the upstream regulators of NUTM2A‐AS1 in NB cells, we utilized the NCBI (https://www.ncbi.nlm.nih.gov/), UCSC (http://genome.ucsc.edu/) and JASPAR (http://jaspar.genereg.net/) databases. Our exploration identified NR1D1 as a potential regulator of NUTM2A‐AS1. Subsequently, we established cell models by stably introducing NR1D1 siRNA into NB cells, and the transfection efficiencies were confirmed (Figure 8A). The results demonstrated that NUTM2A‐AS1 expression is positively regulated by NR1D1 in NB cells (Figure 8B). Predicted binding sites between NR1D1 and the NUTM2A‐AS1 promoter regions were indicated (Figure 8C,D). The ChIP assay further validated the direct binding of NR1D1 to the NUTM2A‐AS1 promoter in NB cells (Figure 8E). Subsequent luciferase reporter gene assays revealed that NR1D1 directly binds to the P4 region of the NUTM2A‐AS1 promoter (Figure 8F). These collective findings support the notion that the transcription factor NR1D1 plays a crucial role in regulating NUTM2A‐AS1 expression in NB cells.

## 4 DISCUSSION

The remarkable efficacy of cisplatin in cancer therapy is, unfortunately, hampered by the frequent emergence of a chemoresistance phenotype in cancer cells. This resistance is attributed to a multitude of factors, including diminished blood flow into the tumour mass, compromised internalization cisplatin, heightened exocytosis of the drug, intracellular sequestration of cisplatin, impairment of DNA repair mechanisms, thwarting of apoptosis‐inducing pathways and the presence of quiescent, non‐cycling cells existing in a non‐growing or non‐dividing state for prolonged periods. These intricate and diverse mechanisms collectively contribute to the formidable challenge of overcoming cisplatin resistance in the context of cancer treatment.^25^ Cisplatin stands as a pivotal chemotherapeutic agent in the clinical management of neuroblastoma,^26^ exerting its effects by targeting DNA through the induction of DNA adducts and cross links. This mechanism leads to the initiation of single‐ and double‐strand breaks, activating the DNA damage response and ultimately triggering apoptotic cell death through both intrinsic and extrinsic apoptotic pathways.^27^ Regrettably, the development of acquired cisplatin resistance poses a substantial challenge, diminishing the effectiveness of clinical therapy in neuroblastoma patients and contributing to instances of chemotherapeutic failure and tumour relapse.^28^ Delving deeper into the molecular dynamics of cisplatin resistance in neuroblastoma is essential for elucidating the complex pathways that confer this phenotype. Such research is pivotal for pinpointing new, impactful therapeutic targets that can improve patient outcomes and propel forward the treatment modalities for neuroblastoma. Our investigation revealed a significant upregulation of NUTM2A‐AS1 in cisplatin‐resistant NB cells, with its knockdown rendering the cells more susceptible to cisplatin. Additionally, the suppression of NUTM2A‐AS1 curtailed the tumorigenic capabilities of NB cells. These findings underscore NUTM2A‐AS1's dual role in facilitating cisplatin resistance and acting as an oncogene in NB progression, thereby spotlighting its potential as a target for therapeutic intervention.

RNA‐binding proteins (RBPs) constitute a class of proteins that engage in specific interactions with RNAs, forming ribonucleoprotein complexes.^29^, ^30^ The interplay between lncRNAs and RBPs serves as a crucial mechanism by which lncRNAs modulate their biological functions.^31^ LncRNAs can function as scaffolds or decoys, either enhancing or attenuating the interactions between RBPs and various biological macromolecules, such as DNA, RNA and proteins.^32^ To understand the molecular mechainsms underlying the biological role of NUTM2A‐AS1 in NB, we performed RNA pulldown coupled with MS assays. Results indicated that B7 homologue 3 (B7‐H3, also called CD276) as a promising associated protein with NUTM2A‐AS1 in NB cells. B7‐H3 has become a focal point for scientific inquiry due to its significant role in various cancer‐related processes, encompassing tumour metabolism, angiogenesis, invasion and therapy resistance.^33^ While B7‐H3 exhibits crucial role in multiple cancer progression.^34^, ^35^, ^36^ The dual role of B7‐H3 in the immune system has given rise to debates, with some studies suggesting its activity as a co‐stimulatory factor for T‐cell activation, while others propose its role as a molecule that primarily functions to downregulate T‐cell‐mediated immune responses.^37^, ^38^ Our research has elucidated the complex role of NUTM2A‐AS1 within the biological framework of NB, particularly highlighting its critical function in modulating B7‐H3 protein stability by preventing its ubiquitination in NB cells. Additionally, our exploration into B7‐H3's biological activities in NB, under both cisplatin‐treated and untreated scenarios, demonstrated that the silencing of B7‐H3 markedly diminishes the IC50 values for cisplatin in resistant NB cells across various concentrations. This finding, in conjunction with the identification of B7‐H3 as an oncogenic factor in NB progression and its involvement in the regulation of NB cell‐mediated immune evasion, reveals the multifaceted influence of B7‐H3 on the disease's pathogenesis.

In our continued investigation into the regulatory mechanisms of NUTM2A‐AS1 in NB cells, bioinformatics analyses highlighted NR1D1 as a putative transcriptional regulator of NUTM2A‐AS1. Following this lead, experimental validations, including ChIP and luciferase reporter assays, substantiated the role of NR1D1 in driving the expression of NUTM2A‐AS1 within NB cells. Notably, NR1D1 expression levels did not vary in response to different concentrations or durations of cisplatin exposure, suggesting a complex interaction that merits deeper exploration to fully comprehend NR1D1's function in NB pathophysiology.

Our research provides significant insights into the roles of NUTM2A‐AS1 in neuroblastoma (NB), highlighting its contributions to cisplatin resistance, oncogenic activities and modulation of immune evasion mechanisms in NB cells. The elucidation of NUTM2A‐AS1's interaction with B7‐H3 and its transcriptional regulation by NR1D1 uncovers complex molecular dynamics underpinning cisplatin resistance in NB. These findings lay a groundwork for subsequent studies focused on the development of targeted therapeutic strategies for neuroblastoma, aiming to improve treatment outcomes and patient prognoses.

## AUTHOR CONTRIBUTIONS

**Tian Xiang:** Data curation (equal); investigation (equal); software (equal); validation (equal); visualization (equal). **Yejing Li:** Data curation (equal); investigation (equal); software (equal); validation (equal); visualization (equal). **Gao Liu:** Conceptualization (equal); funding acquisition (equal); methodology (equal); project administration (equal); resources (equal); supervision (equal); writing – original draft (equal). **Xianyun Li:** Conceptualization (equal); funding acquisition (equal); investigation (equal); project administration (equal); resources (equal); supervision (equal); writing – original draft (equal).

## FUNDING INFORMATION

This work was supported by the National Natural Science Foundation of China (82060539 and 82360477), Natural Science Foundation of Hubei Province of China (2022CFB344) and the Scientific and Technological Project of Enshi Tujia and Miao Autonomous Prefecture of Hubei Province (D20220059).

## CONFLICT OF INTEREST STATEMENT

The authors declare that they have no competing interests.

## Data Availability

The data present in this study will be obtained upon reasonable request from authors.
